# Surface Defect System for Long Product Manufacturing Using Differential Topographic Images

**DOI:** 10.3390/s20072142

**Published:** 2020-04-10

**Authors:** F.J. delaCalle Herrero, Daniel F. García, Rubén Usamentiaga

**Affiliations:** Department of Computer Science and Engineering, University of Oviedo, 33204 Gijón, Asturias, Spain; dfgarcia@uniovi.es (D.F.G.); rusamentiaga@uniovi.es (R.U.)

**Keywords:** defect detection, computer vision, rail surface, long products

## Abstract

Current industrial products must meet quality requirements defined by international standards. Most commercial surface inspection systems give qualitative detections after a long, cumbersome and very expensive configuration process made by the seller company. In this paper, a new surface defect detection method is proposed based on 3D laser reconstruction. The method compares the long products, scan by scan, with their desired shape and produces differential topographic images of the surface at very high speeds. This work proposes a novel method where the values of the pixels in the images have a direct translation to real-world dimensions, which enables a detection based on the tolerances defined by international standards. These images are processed using computer vision techniques to detect defects and filter erroneous detections using both statistical distributions and a multilayer perceptron. Moreover, a systematic configuration procedure is proposed that is repeatable and can be performed by the manufacturer. The method has been tested using train track rails, which reports better results than two photometric systems including one commercial system, in both defect detection and erroneous detection rate. The method has been validated using a surface inspection rail pattern showing excellent performance.

## 1. Introduction

Current industries need efficient quality control systems to ensure their products are free of defects. Because of this, manufacturers invest large amounts of money in developing new inspection systems to control the quality of their products or in acquiring some of the commercial systems available.

Due to the importance of quality inspection, many papers have been published in recent years about the surface inspection in the industry. Computer vision techniques are widely used in many fields [1] as they provide good results in different materials such as fabric [2] or steel [3]. This task is commonly carried out in two steps: first, the surface is acquired using different types of sensors and then the acquired information is processed in order to detect anomalies that are considered defects or which could lead to one.

In this paper, the steel industry will be used as an example, specifically the production of rails for train tracks. The steel industry is a highly competitive market; steelmakers need to improve their inspection systems or develop new ones in order that they can control the quality of the products meeting the tolerances defined by international standards. Therefore, the rest of the producers must improve their quality control systems to remain competitive [4]. Some important factors in the quality of rails are the surface, dimensions and flatness. The inspection of the rails according to these aspects is usually carried out using automatic systems to help the inspectors [5]. Compared to an automatic system, humans are slower and less efficient due to fatigue. In the case of surface inspection, the task is tedious and repetitive, so human inspectors get fatigued or distracted more easily. The cost of having several shifts of inspectors working is high [6]; and while human operators have more sophisticated decision-making capabilities than a machine, their criteria may vary.

Many techniques can be used to acquire a representation of the surface of a product. The most common ones include a camera that takes images of the surface. These approaches vary mainly in the type of illumination. In the case of long products, all of them have one common feature: they place a group of sensors around the piece in order to acquire images of the complete surface of the product.

The traditional techniques consist of acquiring 2D images of the surface using white light sources. The resulting images can be used directly for defect detection based on the color values of the pixels [3], textures [7], or the shape of the piece [8]. These techniques can also be applied to the detection of non-volumetric defects such as discolorations: in the case of steel surfaces, a change in color may be produced by internal defects or material change. However, these methods cannot be used to give accurate measurements of the detected defects. Therefore, these methods simply propose regions that other systems or inspectors must measure before rejecting or accepting the product.

As most surface inspection systems focus their detection process on volumetric defects [9], the 3D acquisition of the product is used. There are two main techniques that generate a 3D representation: photometric stereo [10,11] and 3D laser profiling [12].

Photometric stereo techniques place several light sources in such a way that they produce shadows on the surface in the presence of a volumetric defect. When the position of the light source, the camera and the object are known, the elevation (or depth) of the volumetric defect can be calculated with geometric operations. One of the photometric techniques is called shape from shadow, which includes the Spectral Image Differentiation Procedure [13]. This technique produces images that are processed by a specific algorithm that interprets the color deviations in the images [9]. However, as these methods strongly rely on the light sources, they can be affected by natural light conditions. They are also affected by vibrations because the exact position of the pieces is needed to execute the algorithm. These methods can measure volumetric defects using the shadows they produce on the surface but they lack accuracy. As seen in [9], some techniques such as the Spectral Image Differentiation Procedure can detect changes on the surface from one scan to another but these cannot be compared to the desired shape of the product.

3D profiling systems are based on camera-laser pairs that acquire profiles, which are cross-sections of the inspected piece. These profiles consist of a set of points called point clouds that define the surface of the piece in a section. By combining multiple profiles, a reconstruction of the piece is created. These systems are not affected by natural light because the cameras only acquire the laser wavelength. In many cases, this technique is applied in the manufacturing industry to obtain a complete reconstruction of an object using robotic arms [14] or by taking transversal sections of a piece while either the group of sensors or the piece are being moved [15]. In most cases, the aim of the systems is to measure specific features of the products, such as volume [16] or roughness [17]. Once the surface is acquired, there are two approaches depending on the application: compare the whole surface with the final desired product or compare each profile with a section of the desired product. A long product is defined by its cross-section so each acquired profile can be compared with the cross-section model of the desired product to calculate the differences [18]. When the piece is not defined by a single cross-section, the whole surface must be compared at once, as in the case of circuit boards.

Once the surface is acquired, there are two approaches for processing the resulting images: traditional image processing algorithms and convolutional neural networks. Traditionally, the images are processed using different computer vision algorithms that rely on smoothing the image, looking for anomalies or desired shapes and then filtering the resulting areas based on features such as dimensions, textures, etc. This approach requires knowledge about both the images and the algorithms [3,9]. There is also a final step of classification using artificial intelligence in which ANN or SVM are commonly used.

Many papers have been published in recent years about image processing using only artificial intelligence techniques for defect detection [19,20,21]. The main idea is to use the acquired images as raw input to a Convolutional Neural Network (CNN) or Region-based Convolutional Neural Network (R-CNN) to classify them into defective or non-defective. In this case, the developers must configure the topology of the net and split the huge amount of data needed into several subsets to train, test and validate the algorithm. This approach produces good results, but cant assure that the nets classify according exactly to the defect tolerances defined by the international standards such as the ones in EN13674-1 [22]. This approach is widely used in many fields as it produces good results when the desired regions are well characterized and there are many training instances available. Examples of these methods can be found in several fields such as printed circuit boards [23], casting products [24] or concrete columns [25].

Most commercial solutions to surface inspection give a qualitative diagnosis of the products without taking into account the requirements they must fulfill. This way, quality engineers of the manufacturer must measure the defects themselves in a second inspection step in order to check if they meet the requirements or not. In contrast, the proposed method gives a quantitative measurement of the detected regions from a 3D representation of the inspected product by using several 3D profilometers. This method produces topographic maps of the surface that are represented as images. These images also allow the usage of the international standards as the basis of the detection procedure, using the tolerances they define. This ensures that the system will not classify anything that fulfills the requirements of the international standards as a defect. Likewise, anomalies with greater dimensions than the tolerances of the standards will be classified as defects.

Commercial solutions are also hard to configure by the manufacturer, so the quality inspectors need the assistance of the engineers of the seller companies in order to configure their systems. This is a very expensive and cumbersome task that may last for months in order to get enough data to give an accurate diagnosis. Using the proposed solution the engineers of the manufacturer can configure the system by themselves as it includes a systematic configuration procedure. This procedure is composed of very easy steps and does not need any special hardware. This allows the manufacturer to adapt the method to its production line without cost.

Commercial solutions usually store the information as images of the product in a three-channel format which leads to an enormous amount of needed space. These images can only be used as documentation of the product. Few systems store actual 3D information in raw format, which also increments significantly the amount of data to be stored. In contrast, this proposed method stores the data as one channel images that represent in a differential way the surface of the product. These channel images are an efficient way to store information about the surface and make it possible to use common programs to visualize them. They are also used to detect surface defects and measure them using computer vision techniques. As a result of the acquisition method, these images can be used directly to measure defects as they have a direct translation to real-world dimensions.

The remainder of this paper is organized as follows: the proposed approach is described in detail in Section 2. In Section 3 extensive test results are presented in order to detect defects in the rails used as an example. Finally Section 4 details the conclusions of the paper.

## 2. Proposed Approach

The proposed inspection system is composed of two subsystems: acquisition and detection. The acquisition system acquires profiles from the product using four profilometers and generates a topographic image of the surface of the product. The detection system processes the generated images following a traditional pipeline that takes into account the resolution of the images and the international quality standards. The last step of the detection algorithm is based on a multilayer perceptron that is used to classify the detected regions into defects or non-defects.

The proposed method can be applied to any long product and the information given by the method can be used to implement other applications such as dimensional inspection [26] or flatness inspection [27]. Each type of product must be defined by its model. The model is a set of arcs and segments that define the shape of the desired profile. This concept will be used later in order to explain the acquisition procedure.

### 2.1. Acquisition

**Figure 1:**
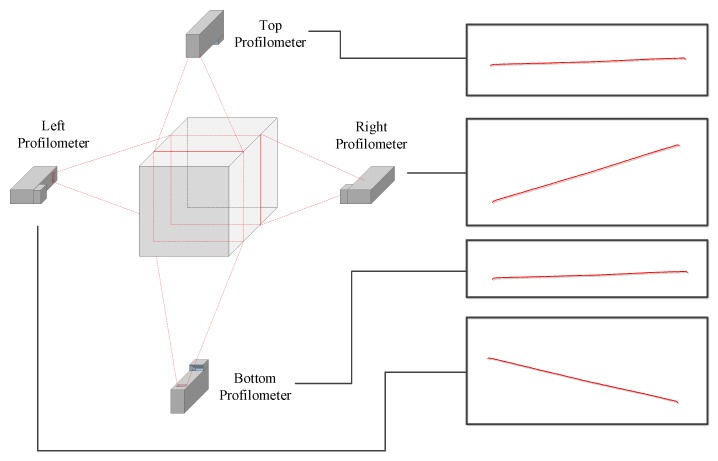
Profile acquisition.

In most cases, long products must be inspected completely, including their whole surface, in order to ensure their quality. In order to acquire images of the whole surface of a product, a set of sensors must be located around it. In this approach, four profilometers are placed around the product. A profilometer is a sensor made up of a camera and a laser. Each of these sensors acquires images of a part of the product defined by a point cloud as shown in Figure 1. The number and the place of the sensors must be set in such a way that the whole contour of the profile can be acquired. The sensors must also be placed so they acquire most of the surface perpendicular in order to acquire correctly the small cracks without occlusions.

The four sensors are placed in two different measurement planes in order to avoid interferences between them while they are acquiring the projected laser line over the product. These interferences occur because the four profilometers project the laser using the same wavelength. The acquisition of profiles by the set of sensors is synchronized using a signal that is triggered using an encoder signal. The encoder is placed in the production line so it sends a tick every 2 mm while the product is being moved.

Once each part of the profile is acquired, the model is used to combine them. Each point cloud is aligned to a part of the model. This procedure is called registration. A partial model is defined as the set of arcs and segments, called primitives, that can be seen by a sensor. Therefore, four partial models must be defined in this example as the number of sensors is four.

The registration procedure is carried out in two phases: first, a coarse registration is done by aligning the centroid and the orientation of the point cloud and the partial model, then a fine registration is done using the ICP algorithm.

After the registration procedure, the differences between the model and the acquired point cloud are calculated. For each point in the point cloud, its closest point in the model is calculated and called the corresponding point. A coordinate origin in the profile contour must be defined in order to represent the position of each point in the contour by a single number, the arc-length from the origin to the point. If the application is suitable to be used for many types of the same product, this origin point must be set carefully so it has the same meaning in every type of profile.

**Figure 2:**
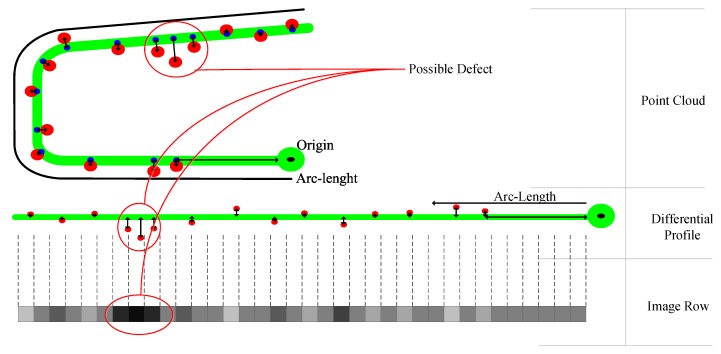
Acquisition and representation scheme.

Then, each point is transformed into a new one that represents the position of its corresponding point in the profile and its distance between the point and its corresponding point. Then, the new point cloud is defined by points that have two coordinates: the arc-length of the profile from the origin to the corresponding point and the distance from the point to its corresponding point. The result of this step is called the differential profile, as shown in Figure 2. In this figure the acquired points, in red, are compared with their corresponding points in the model, in blue. The translation into the differential profile is shown calculating the distance between the point and its corresponding point (red–blue) and the arc-length between the corresponding point and the origin (blue–black).

### 2.2. Representation

The differential profile from each profilometer can be merged into a single differential profile that can be used as a row of an image. Each pixel has two dimensions in millimeters depending on the resolution of the sensor and the acquisition frequency. The whole image can be seen as a differential map of the surface of the product.

Each row of the output images is the result of merging the four differential profiles that form each complete profile. Therefore, the dimension that each row represents in millimeters is set by the acquisition frequency of the sensors. In the same way, the dimensions on the columns axis must be set according to the resolution of the sensors.

The value of each pixel is set so it has a direct translation in millimeters to real-world dimensions. To do so, the measuring range must be translated into the [0,255] range defined by an 8-bit image. There may be some pixels with no associated data depending on the position of the points on the contour of the product. These spots with no data are represented with an exact zero value on the differential map that will be translated into a 127 value when formatting the image. These images can be viewed with a common program, so both inspectors and customers can access the raw information. As the gray value of the pixels can be directly transformed into millimeters, the images can also be used to measure defects and other objects.

This format allows the storage of dimensional information in an efficient way. The format has several advantages: the images can be viewed easily by inspectors and customers, the defects can be measured directly in the images by looking at the gray value, and the detection can be carried out using common computer vision algorithms that use an image as input.

The procedure of acquiring and representing a profile from a point cloud to an image is shown schematically in Figure 2. This figure shows how a defect can be represented in the point cloud, the differential map and also in the image. This figure also shows some spots with no-data that are formatted with 127 value. Taking into account the measuring range, each gray value represents 0.03 mm. This resolution should be enough to be used in many types of products. One of the products with high-quality requirements is train track rails, which must meet surface tolerances up to 0.35 mm, as is set in the European Standard [22] for defects in the head of the rail.

**Figure 3:**
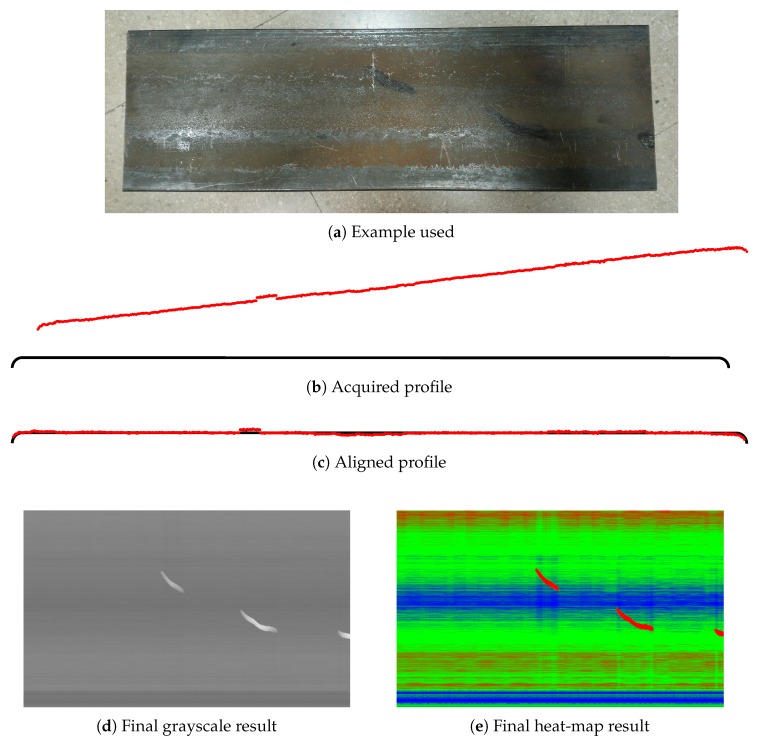
Acquisition and representation example.

The acquisition procedure and the representation is studied in detail in [18]. As an example, in Figure 3 a flat steel surface is shown with the acquisition of one defective profile, its registration and the representation of the surface in the original gray-scale images and another format using heat-map colors. On this surface, there are several defects: there are three protrusions on the surface, marked with black ink, and some dimensional defects including a concavity on the surface which is a bit concave in the middle.

### 2.3. Detection

**Figure 4:**
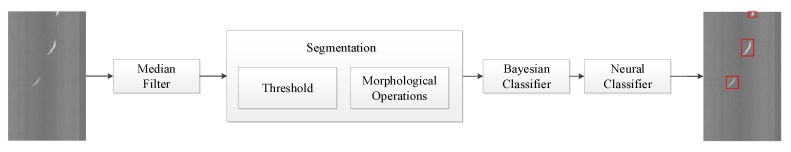
Detection pipeline.

Once the images are acquired they must be processed in order to detect defects. In this approach, a pipeline using computer vision techniques is used, shown in Figure 4. In the first step, the image is filtered to remove the background. Second, a segmentation procedure extracts the anomalies. Next, a Bayesian classification uses expert knowledge of the product to remove erroneous detections in some areas of the image. The pipeline concludes with a final classification using a neural network.

The generated images are topographic maps of the surfaces. This is a representation of the surface using its deviations from the model. However, there are several circumstances that may be considered to filter the images and detect defects. A dimensional defect is defined as a different measurement of the dimensions of the product with respect to the model. This is caused by a lack of material in the source piece at the moment of rolling it into its final shape. Although dimensional defects may be visible on the surface of the product, they are not surface defects. Conversely, the surface of these areas may be perfect even when the product does not fit the desired dimensions. Because of the acquisition procedure, a dimensional defect may appear in the images as a continuous defect along with the product. Another problem are noisy zones that appear because some parts of the product may be not perpendicular to the sensors. In these areas the laser line is not acquired in an ideal manner, producing bigger variations in the measurements with respect to the perpendicular zones due to the reflection of the laser on oblique surfaces. These two problems cause areas in the images with two characteristics: continuous deviations along the length of the product and random noise.

The international standards about surface quality define a defect as a minimum deviation (in height or depth) that an anomaly must have in order to be considered a defect. As the value of the pixels represents this deviation, the minimum tolerance defined in a standard can be used as a threshold value in a segmentation process. However, the images must be further processed before the segmentation in order to mitigate the effects of the problems mentioned above.

#### 2.3.1. Median Filter

The main objective of performing a median filter on the image is to make the image a differential map by extracting its background. In the image a spot with no acquired points, this is no-data spot, is defined as having a value of 127. This value is an exact zero value and is statistically unlikely to appear as a measured value. In fact, zero values should be in a range near 127 value as each gray value represents 0.03 mm.

As each column defines the same position in the contour of the product along its length, the filtering is performed by column. For each column, the values which are different from the no-data value are taken and their median value is calculated. Then, the median is subtracted from the original values in order to get a new image centered on zero.

Once the image is filtered, the dimensional defects should have been eliminated, but the noisy regions are still present in the image. Thus, further filtering may be needed in the next steps.

**Figure 5:**
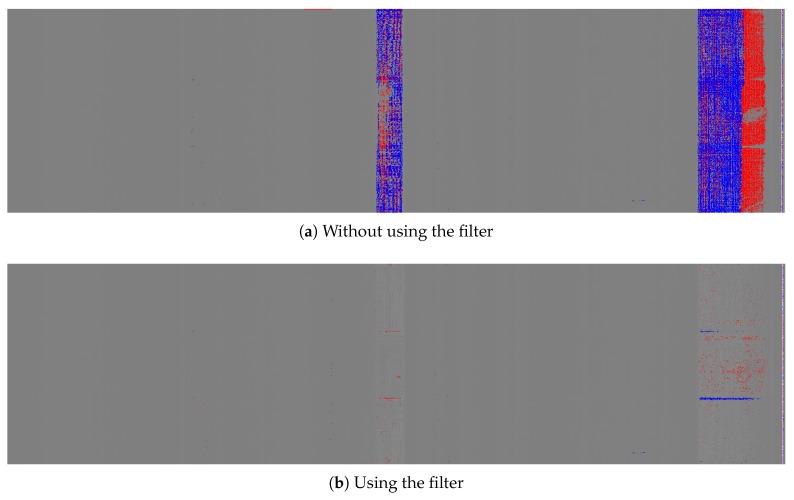
Filter effect.

In order to illustrate the problem generated by dimensional defects and the effect of the filtering, two images are presented in Figure 5. In these images, two-dimensional defects appear in two zones. These images are processed in order to obtain the pixels of the images that represent a deviation that may be a defect. The image in Figure 5a has not been filtered before the processing. It shows defective pixels in color: blue for negative deviations and red for positive deviations. The image in Figure 5b was filtered before the processing showing that the dimensional defect is no longer detected as a surface defect.

The median filter is carried out by column. This filtering removes dimensional defects and subtracts the background at the same time. Surface defects are maintained as they represent a deviation from the background, which is the perfect shape in the ideal situation but it can also be a dimensional defect. One example of surface defect on a dimensional defect can be seen in Figure 3d,e, where a dimensional defect appears in the middle zone of the flat surface while some surface defects are also in that zone.

#### 2.3.2. Segmentation

After filtering the main objective is to segment the anomalies on the surface. An anomaly is defined as anything which differs from the perfect surface. An anomaly is classified as a defect when its deviation is greater than a value defined by the international quality standards. The segmentation procedure is divided into two steps: thresholding and morphological operations.

Production conditions can be taken into account in order to improve the detection. The production process of each type of product is different so the production conditions may vary the way the segmentation can be optimized. For example, if some parts of the products have no surface quality requirements, they do not need to be segmented, avoiding the detection of erroneous regions. This optimization is completely dependent on the production procedure.

Thresholding is applied based on the tolerances defined by the international quality standards. Therefore, any anomaly that surpasses the tolerances will be segmented by the thresholding. A standard can define many tolerances for many zones of the product. Depending on the production conditions it could be better to apply the strictest tolerance to all the zones so defects that are in more than one zone can be completely segmented.

After thresholding, two morphological operations are used [28]: opening and closing. An opening is an erosion followed by a dilation and a closing is a dilation followed by an erosion. These operations require a mask to be used around the contour of the regions. An erosion eliminates the pixels of the region that are inside the mask when it is moved around the contour of the region. A dilation adds the pixels that are inside the mask when it is moved around the contour of the region. Therefore, an opening tends to eliminate small regions because small regions that are completely eroded cannot be dilated later. On the other hand, a closing tends to join adjacent regions because they are joined thanks to the first dilation.

In this step, noisy regions can be characterized as columns or a small group of columns that have a large number of pixels above the threshold (or below in the case of depth). These noisy regions are produced due to the noise introduced by the oblique acquisition of some parts of the surface, depending on the shape of the product. To eliminate them, a mask is defined as a long rectangle to be used in a closing followed by an opening. Then, only white noise, defects and other anomalies such as dirt and scales are left as the segmented regions.

After eliminating noisy regions, the main objective is to eliminate the rest of the noise and small scales, and to join all the pixels that represent a defect. To do so, two other masks are defined to be used in an opening operation and a closing operation respectively. The order of these operations is set so the small unwanted regions can be eliminated with the opening before the pixels are joined using the closing. The closing operation is also used for filling holes inside the regions.

**Figure 6:**
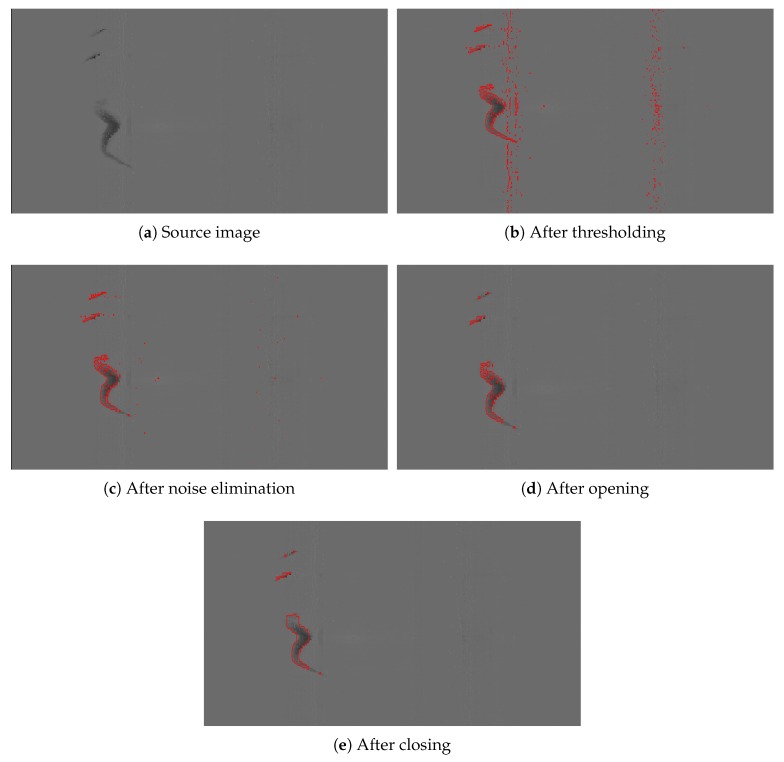
Segmentation procedure.

This segmentation procedure is illustrated in Figure 6 using an image with a defect as an example. In this case, the segmented regions are shown in red after each step of the procedure.

#### 2.3.3. Bayesian Classifier

The segmentation step generates a set of regions that can be seen as potential defects. In this step areas with a large amount of dirt and scales have produced several regions that are not defects and must be eliminated. Expert knowledge can be used in order to eliminate some of these regions before further steps of classification. In order to do so, the experts can say which areas are the ones with more dirt, water or scales that can be seen as a volumetric anomaly that is not really a defect. The depth/height of the regions segmented in those zones is studied, but this step may change depending on the industrial context, the type of product and the morphology of the defects.

**Figure 7:**
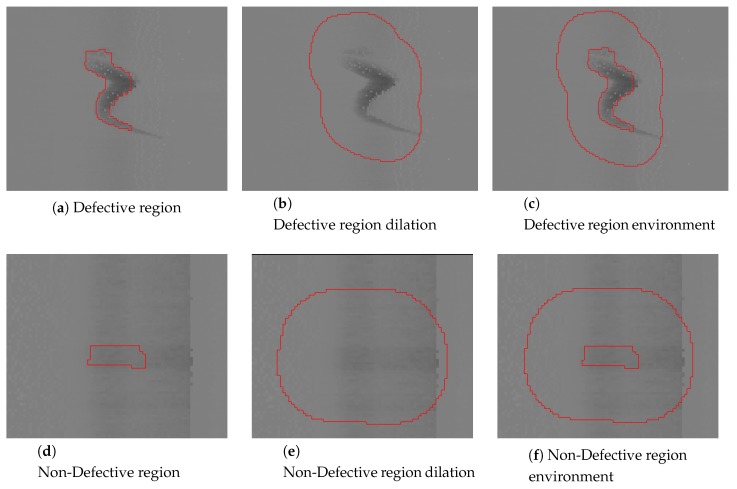
Region environment extraction.

The main objective of the step is to analyze the potential defects and their environment. To do so it is important to obtain the features of the regions and their environment. The environment of a region is defined as a dilation of the region in which the region is not included. Figure 7 shows an example using the defect viewed in Figure 6 and an example of a segmented region that is not a defect.

Once the environment is extracted, the number of pixels above and below the used threshold is calculated in the region and the environment, and they are compared with the size of the region and the environment respectively. The extracted data is analyzed in order to apply the Bayesian decision theory [29]. To filter the non-defective regions, a limit value must be set. The feature extracted from both, region and environment, has different distributions for defective and non-defective regions. These distributions can be analyzed in order to set this limit value. The distribution of the difference between the region and the environment can also be studied.

#### 2.3.4. Neural Classifier

The last part of the method is based on machine learning techniques. The configuration of a method based on ad-hoc rules is complex and difficult to automate the configuration. Furthemore, if the method relies completely on ad-hoc rules, it will be more unstable and not adaptable. Thus, a machine learning approach is used as a final step of the method in order to be more flexible and automatically configurable. This last step is composed of a neural network used as a binary classifier. This network filters erroneous detections, but it can also be used to eliminate some other volumetric anomalies that are not surface defects such as engravings or junctions.

In order to train the neural network, 44 features are obtained from each instance. These features are divided into two sets [30]. The first set of features defines the morphology of the region. This set includes 14 features as: gravity center, length and width, area, center of the bounding box using a rectangle or an ellipse, etc. This first set gives information about where the region is located and what shape it has. This is suitable for regions that are likely to appear in particular zones as scales, dirt or noisy zones depending on the image acquisition.

The second set of features contains 30 features about the values of the pixels in the region. This second set defines the appearance of the region in order that it can be differentiated from some erroneous detections that could be similar. Some examples of these features are: standard deviation of the values, entropy, anisotropy, contrast, etc. This set also includes as features the parameters resulting from approximating the region to a plane and a second-order surface.

**Figure 8:**
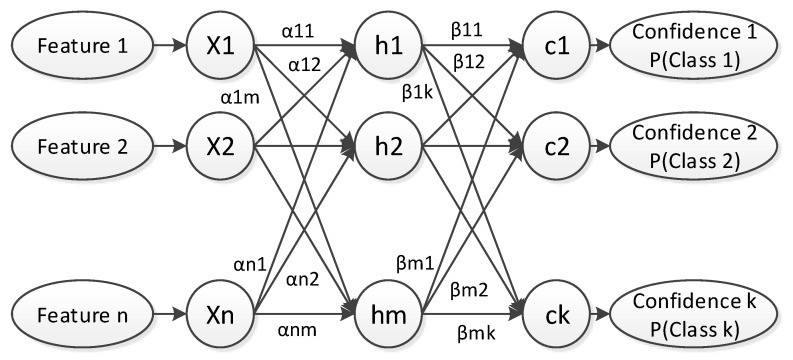
Multilayer perceptron.

The neural network used is a multilayer perceptron, as shown in Figure 8, that takes as input the 44 features described. This network uses a hyperbolic tangent as the activation function in the hidden layer (1), where matrix α represents the weights of the input layer and vector $b^{1}$ represents the weights of the bias.

(1) $$h_{j}=tanh(\sum_{i=1}^{n}\alpha_{i,j}x_{i}+b_{j}^{1}),j=1\ldots m$$

A normalized exponential function is used for obtaining the values of the hidden layer, (2) and (3). In these equations, the matrix β represents the weights of the hidden layer and vector $b^{2}$ represents the weights of the bias.

(2) $$a_{j}=\sum_{i=1}^{m}\beta_{i,j}h_{i}+b_{j}^{2},j=1\ldots k$$

(3) $$c_{l}=\frac{e^{a_{l}}}{\sum_{t=1}^{k}e^{a_{t}}},l=1\ldots k$$

The third layer of the network, the output layer, has k nodes. The output of these nodes is the confidence of a region belonging to each class (defect or erroneous detection). Therefore, there are as many output nodes as classes, 2 in this case. The sum of these two outputs must be 1. The class with the highest confidence will be used to classify each region.

In the set of 44 features described, there can be some that are not suitable to be used for classification in some circumstances. The best features to classify the regions must be selected. Thus, a Principal Component Analysis (PCA) is used to select the best features. The output of the PCA is used as input for the neural network in order to improve its performance. From all the features only the *n* most relevant for the classification will be used. The number of principal components to be used as input is a parameter to be configured.

The network is trained until one of the following conditions is met:

- 100 iterations have been performed
- The change of the sum of all weights between iterations is less than 1
- The change of the error value between iterations is less than 0.01

### 2.4. Parameter Configuration

The parameter configuration of many inspection methods needs special hardware, such as GPU to train CNN. The proposed method can be carried out using common computers in an affordable amount of time.

Several parameters must be configured in order to use the proposed method. These parameters are: segmentation threshold, opening and closing segmentation masks, bayesian classifier parameters and the neural classifier parameters. This configuration is done following the pipeline defined by the detection method so the output of a configured step can be used as an input to configure the rest.

The segmentation step is configured using a factorial design. To do so, a set of possible configurations must be obtained to test their performance. These possible configurations are obtained by combining the possible values of each parameter. Each parameter must have a set of possible values that must be selected according to their physical meaning. In the case of segmentation threshold, for example, the value must be set in order to meet the depth/height requirements defined by the international standards.

The Bayesian classifier step is configured analytically by extracting and studying the features of the previously obtained regions. The environments of these regions are also analyzed. Features extracted from the regions and their environment will have a unique distribution that can be studied in order to find a discrimination value.

The neural classifier is configured through an exhaustive exploration of the parametric space. This step has only two parameters: the number of inputs after the PCA and the number of hidden nodes. As a general rule, this exploration can be set from 1 to the number of features in each parameter.

To configure the parameters of the method, a metric to evaluate the adequacy of the values given to the parameters is needed. In this case, the metric estimates the goodness of a detection comparing it with a “perfect detection” which comes from a Knowledge Base (KB). The KB used must be composed of enough products to represent the production of the manufacturer and the common defects produced. Each product in the KB should be inspected manually by an expert inspector to ensure that every single defect is well labeled.

The evaluation is done as a comparison between the KB and the result of the method. A true-positive (TP) is defined as a detected region that intersects with a defect labeled in the KB. In the same way, a false-positive (FP) is defined as a detected region that does not intersect with any defect labeled in the KB. Each defect labeled in the KB is defined as a false-negative (FN) if it does not intersect with any detected region. Thus, the best performance is obtained by maximizing TP and minimizing FP and FN.

The values of TP, FP and FN from the evaluation of a detection must be used to calculate some metric in order to estimate the goodness of the result. These metrics must follow the objectives set by the industrial process: maximize the defect detection and minimize the number of erroneous detections. To do so, two metrics are calculated using the values of TP, FP and FN.

The selected metric to evaluate the erroneous detection rate is the mean of the erroneous detections per product. This metric is obtained by processing the KB. Each false-positive also means an economic loss for the manufacturer because it must be checked by a technician. Thus, the objective must be to minimize this metric.

A similar metric cannot be used to estimate the number of correct detections, because the metric must consider how many defects should have been detected. Therefore, a standard metric called Recall (4) is used to do so. This metric is defined as the relation between what has been detected and what should have been detected. The higher the value of this metric, the better the detection.

(4) $$Recall=\frac{TP}{TP+FN}.$$

Detection of all defects (Recall=1) leads to a large number of erroneous detections (FP>>0). Thus, a compromise must be found.

## 3. Case of Study and Results

In this section, the proposed method will be configured and tested using train track rails as an example of a long product. Clearly, the values given in this section for each parameter may vary for other products and industrial environments and the metrics used could not fit the requirements of some manufacturers. The rail industry has international quality standards, such as the EN13674-1 [22] in Europe, which establish surface requirements that must be fulfilled.

**Figure 9:**
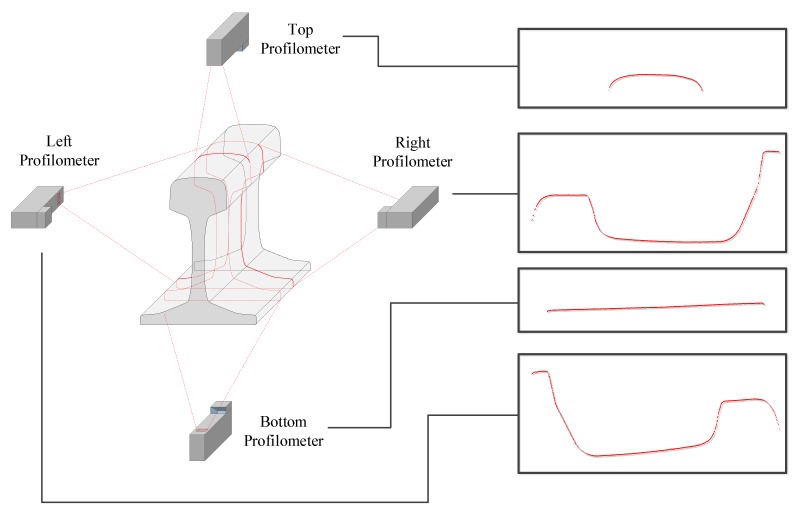
Rail profile acquisition.

First, the positioning of the sensors around the product must be discussed. In Figure 9 the proposed positioning of the sensors for rails is shown. The profilometers are placed in such a way that they acquire most of the surface of the rail perpendicularly. As it is a critical region, the head of the rail (top side) must be inspected perpendicularly in any possible scenario as it is the region that physically makes contact with the train wheel. Therefore, the position of the top profilometer is not variable. In this work, another three profilometers are used in order to acquire the bottom of the foot (which is also a critical region) and both sides of the web of the rail. Using these placements, two laser planes must be used in order to avoid interactions between side profilometers and top/bottom profilometers.

**Figure 10:**
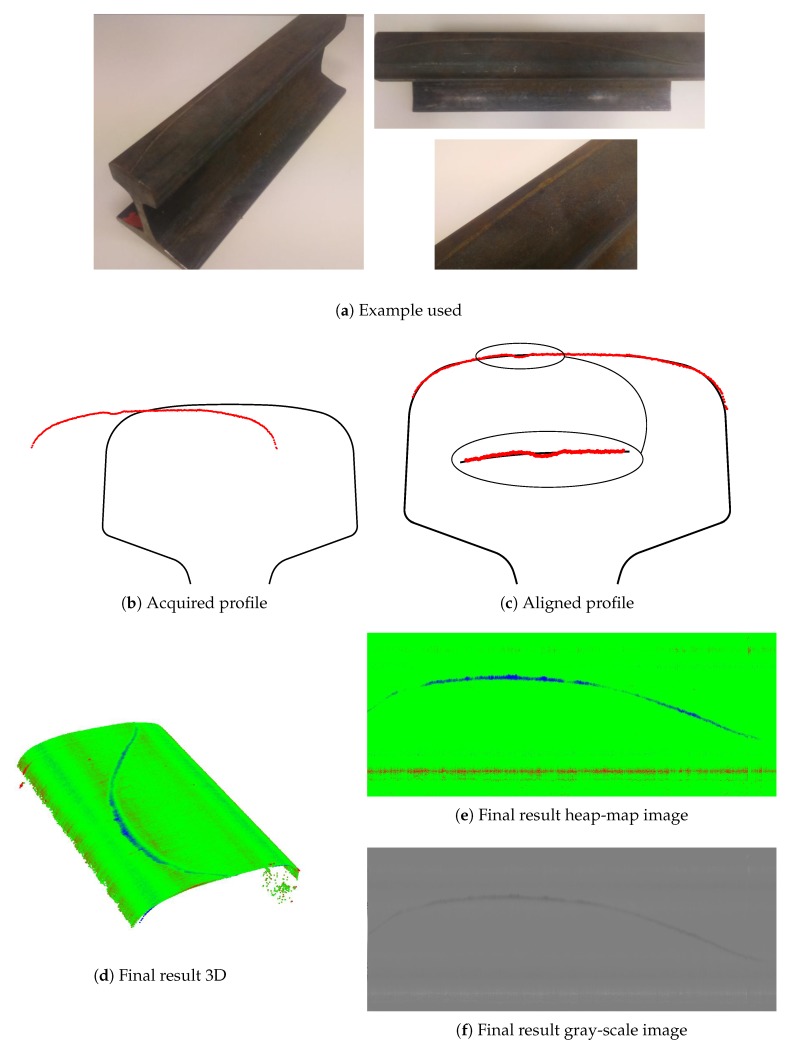
Acquisition and representation example of a real surface defect.

An acquisition example of a defect in a real railhead is shown in Figure 10.

### 3.1. Detection

In order to configure, test and validate the method, a Knowledge Base is generated. This Knowledge Base is composed of images from 65 inspected rails of between 80 and 110 m each, with an average of 50 images per rail. In this knowledge base, there are 105 defects, 55 identification stickers and abundant dirt and scales. Note that the stickers are not defects but they may be detected as if they were. These are real rails manufactured in a real industrial environment so the rails can only be acquired once. These rails are exhaustively manually inspected so the results of the method could be compared with the real defects that exist on the rails. In the production line, there are another two surface inspection systems whose results will be used for comparison.

In order to give better guidelines to the reader, the sections with the description of the steps of the detection method and their correspondence configuration are shown in Table 1.

### 3.2. Median Filter Configuration

The median filter should be configured just in case the surface defects can be as long as the physical length is represented in the images. As it makes the filter by column, it is supposed that any dimensional defect appears in all the length of one image.

In the case of rails, dimensional defects can have more than 2-m length which is the physical length represented by an image. At the same time, common surface defects usually have several millimeters in length, never more than half a meter. This situation makes that filtering the whole column will not affect the surface defects but it will eliminate the dimensional defects.

### 3.3. Segmentation Configuration

The segmentation threshold must be set in such a way that all defects are segmented in this step. International standards set quality requirements that can be used to configure this step, and it is essential that all the defects identified by the production line should surpass the tolerances in these standards. In addition, the sensors used should be tested to establish the intrinsic white noise they introduce in the measurements.

In the case of rails, the strictest quality standard defines a defect as having a deviation greater than 0.3 mm. Thus, the value used in this example for thresholding is 0.3 mm in height and depth. After thresholding, adjacent pixels are joined into regions. In order to check the viability of using the international standard value and to look for the value that gives the best results, a study of the threshold value is performed using the values in the interval [0.15, 2]. The value 0.15 is selected as the starting point because the noise introduced by the sensors is approximately this value [18].

As happens with other products, in rails some zones are defined in which different requirements must be fulfilled. In this particular case, four zones are defined: head, web sides, foot sides and foot. Each of these zones can be processed independently in order to set different qualities or to take advantage of the knowledge of the production process of the product. The strictest quality standard values are used in all zones even in zones for which the requirements are not as strict. In the case of rails, there is a zone on the web sides with cast marks for identification that can be detected as defects. However, the production process ensures that no defective protrusion can appear in this zone other than dirt or scales. Taking advantage of this, in this zone, only anomalies with a negative deviation will be segmented.

**Figure 11:**
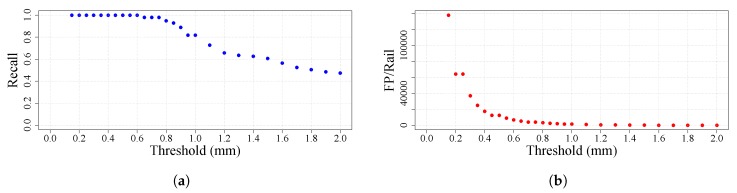
Segmentation threshold exploration. (**a**) is the evolution of the Recall depending on the value of the segmentation threshold. (**b**) is the evoution of the number of False Positives per Rail depending on the value of the segmentation threshold.

The metrics obtained from this study are shown in Figure 11. The international standard value (0.3) proves to be a good value for the threshold because it gives a result in which all the defects are segmented and the false-positives per rail drops sharply. A higher value could lead to missing some defects with a minimum gain in the reduction of false-positives.

Once the regions are segmented, some morphological operations are performed. The mask used in the opening and closing operations must be defined according to the dimensions of the pixels. As the masks used are rectangular, there are four parameters that must be configured: the heights and widths of the opening and closing rectangles.

To configure this, an exhaustive search is done over the parametric space in order to find the best solution. The ranges of exploration must be set in such a way that they make sense in terms of dimensions. For example, it is not appropriate to set a mask of height equal to 20 pixels in an application in which the pixels of the image represents 2 mm. Such values would mean that regions should be put together when they are at 2 cm. Therefore, the values of these parameters must be set carefully in order to keep consistency with the real world dimensions the pixels represent.

**Figure 12:**
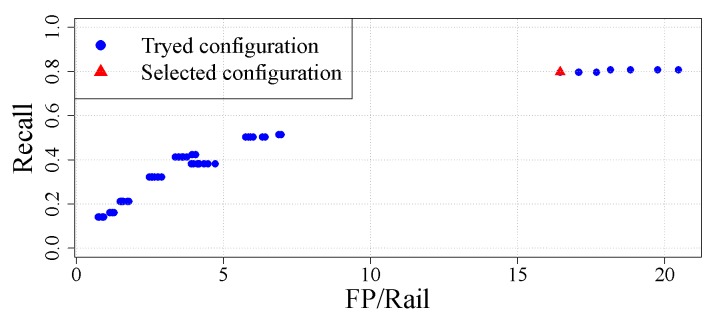
Evaluated configurations for mask dimensions.

By changing all four parameters, 64 configurations are evaluated measuring the recall and the number of false-positives per rail they give. These configurations are shown in Figure 12 in which the selected configuration is shown in red. From all the configurations the ones that are worthy of consideration (those with the highest recall) are in the top right of the figure. Those are the ones with the highest recall. As false-positives will be reduced in later steps, these should be the ones chosen as they maximize correct detections.

### 3.4. Bayesian Classifier Configuration

Once the previous steps are configured, the features of the segmented regions must be gathered in order to analyze their distribution. The procedure used to analyze this is based on a comparison of the histograms [29] from the segmented TP and FP and their environments. This comparison can be done between the regions and their environment, or by directly comparing the regions (TP and FP) or the environments (TP and FP) themselves.

Using expert knowledge given by quality inspectors some erroneous regions can be eliminated. In the case of rails, this step must be done on the sides of the foot of the rail as they are zones with a great amount of dirt and scales. As scales and dirt are the most important source of false detections, the depth/height of the detected regions is studied because scales and dirt are always seen as volumetric anomalies.

Comparing the number of pixels below the depth threshold and above the height, threshold shows a difference between the environments for TP and FP. A limit value for selecting a region can be established at 0.025, which means that any region that has an environment with more than a 2.5% pixels outside the segmentation threshold interval will be identified as a defect. This rule eliminates 40% of the false-positives reducing the minimum number of true-positives.

**Figure 13:**
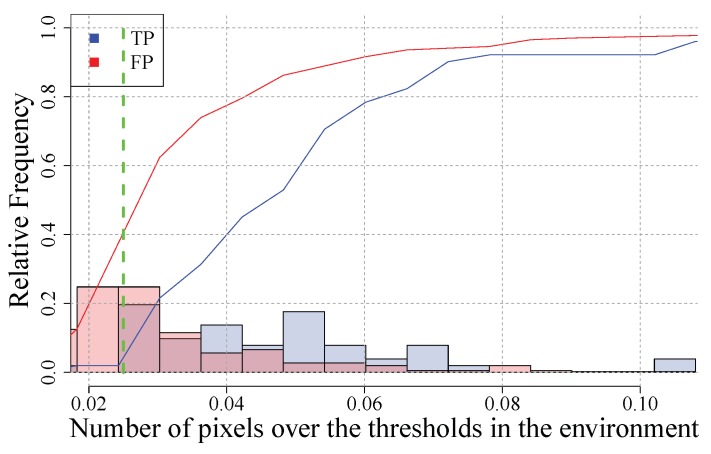
Environment filter distributions. Distribution of the values of the true-positive (TP) in red while distribution of the values of the false-positive (FP) in blue.

The distributions that lead to this bayesian filter can be seen in Figure 13. In this figure, the histograms of both TP and FP are shown in blue and red respectively with 50% of transparency. The cumulative relative frequency is shown too in the same Figure as a line because it is useful for comparing them and set the limit value.

### 3.5. Neural Classifier Configuration

The configuration of the neural network consists of the configuration of the parameters and also the training. This is carried out using the results of the previous steps. The configuration of the parameters includes the number of principal components to be used (from PCA) and the number of hidden nodes. An exhaustive search is performed in this two-dimensional parametric space as it is computationally viable.

The number of nodes in the input layer is the same as the number of features, that is, the number of principal components used. This number varies from 1 to 44. The number of nodes in the hidden layer is selected in some works as the 2-base logarithm of the number of training instances. However, the optimization of this factor is still an unresolved problem [31]. Thus, this factor is studied in the same range as the input layer, from 1 to 44.

The set of instances used for training the network presents a situation of imbalanced classes, which is pretty frequent in classification problems. If the set contains 93% of false-positives, the best training could be classifying all instances as false-positives obtaining a 93% of success rate. There are several methods to fix this problem [32,33]. One of these methods is the random undersampling, which is used in this case. This method selects *N* from each 10 instances for training. In these experiments, *N* is another factor that varies from 1 to 10. Therefore, each combination of the number of principal components, the number of hidden nodes and subsampling rate is evaluated. Each of these combinations is evaluated with the K-Fold Cross Validation method using four folds.

To evaluate each configuration, two metrics are used: true-positive rate (TPR) and true-negative rate (TNR). These are the rate of instances that are well classified from both classes so the best configuration will be the one that maximizes both metrics.

**Figure 14:**
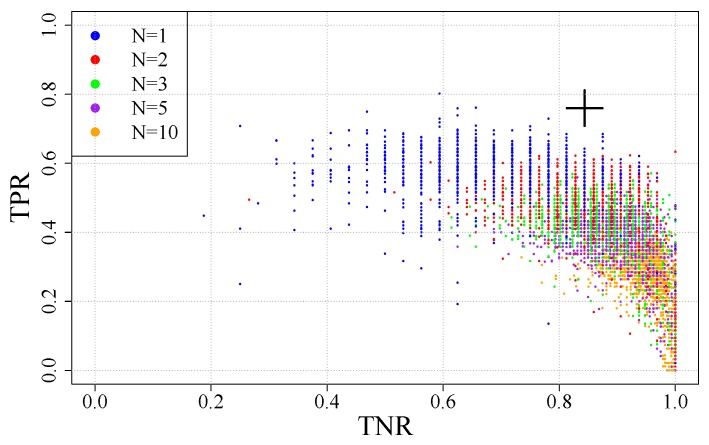
Neural network configurations.

The number of configurations is huge, so in Figure 14 only the configurations from some undersampling values are given in order to show the trend. This figure also shows the selected configuration with a large black cross.

The selected configuration achieves a 0.76 in TPR and 0.84 in TNR, which means that the use of this network will eliminate most of the false-positives while keeping most of the true-positives. This configuration uses 20 hidden layer neurons and 9 principal components for the classification and was trained with 1 from each 10 instances of false-positives, which is approximately 1 to 1 compared with true-positives.

### 3.6. Overall Performance

The proposed approach, configured as described, achieves a Recall of 0.63 with only six false-positives per rail. This result is obtained from the rails in the knowledge base.

The detection method is compared to two surface inspection systems for rails. Both systems, A and B, are based on stereo-photogrammetry. System A is a commercial system whose detection method is described in [13], whereas System B is a published system that follows a similar pipeline to the one used in this paper [9]. The proposed method gives better performance than these two surface inspection systems as shown in Table 2.

This detection is performed in real-time conditions with a limitation of 2 s per image. Using the produced images this means that the proposed system can acquire and process enough information to detect defects in a production line that produces rails at 1 m/s.

**Figure 15:**
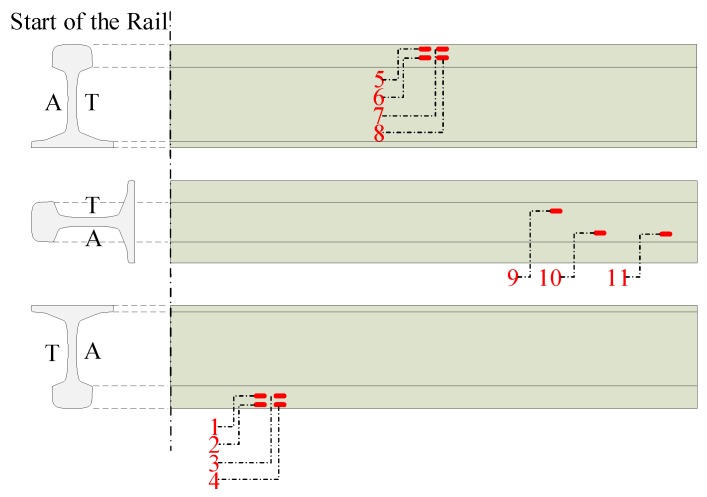
Validation rail pattern.

In order to validate the result, another rail is used. This rail is a pattern used to calibrate and validate surface inspection systems that need to meet an international quality standard. This rail has 11 longitudinal defects intentionally planted on its surface to check the quality of the inspection. The rail pattern is shown schematically in Figure 15 and the dimensions of the defects are shown in Table 3.

From the longitudinal defects, the detection procedure detects 10 TP and 21 FP before the neural classifier step. After that classification, 6 TP are left with only 8 FP detected. As can be seen in Table 3, the not-detected defect before the classifier has a width that is near the resolution of the sensor used, so it can be said that the detection is hardly dependent on the resolution of the sensors used. The TPR achieved by the classifier is validated with an exact rate of 0.6. The achieved recall using the pattern rail is 0.54. Even when these results differ from the ones obtained with the Knowledge Base, the longitudinal defects on the pattern are less than 1 mm in width whereas most of the real defects are much bigger, exceeding several millimeters in width.

**Figure 16:**
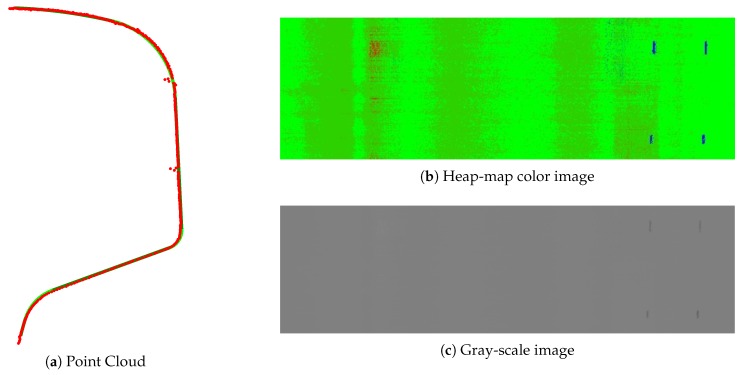
Defects in the rail pattern.

As an example, one of the profiles that contain defects 1 and 2 is shown in Figure 16 with the heap-map colors and gray-scale representation of the generated image.

## 4. Conclusions

Detecting surface defects generated in long products during their production is a very important requirement to ensure their quality. In the case of rails, used as a typical example of a long product, this detection is vital since surface defects in the rails can affect the safety of the train directly.

In this paper, a non-invasive method to detect surface defects in long products is proposed. The main benefits of this proposed solution are listed below:

- No special light conditions required for installation.
- Equipment takes very little space in the production line.
- Efficient information storage.
- Common format images generated can be visualized by any common program.
- Defects can be directly measured over the images thanks to direct mapping to real-world dimensions.
- Systematic and automatic configuration procedure that can be performed by manufacturer technicians requiring no special hardware.
- Detection based directly on the international standard requirements.
- Inspection performed in real-time while the product is moving at the end of the production line so it neither slows down the production nor needs an extra step in the process.
- Acquisition and inspection can meet production speeds of 1 m/s.
- High detection rate compared to existing commercial systems and very low false detection rate.

The generated images can be used to measure the defects directly as they have a direct mapping to real-world dimensions. The acquisition is based on accurate sensors that have a resolution of 0.1 mm. This feature gives the system the chance to detect defects according to the requirements of the international standards, which is a key feature needed in current industries.

The method has been tested on 65 rails with 105 defects. These rails are from 80 to 110 m in length resulting in approximately 6500 m of rail inspected. The images from these rails where acquired with a frequency that gives 1 rail profile each 2 mm resulting in more than 3 million profiles. The execution of the method over these rails detects 63% of the defects with six erroneous detections per rail. Although this detection rate seems low, it makes the erroneous detection rate low too. Detecting many erroneous defects slows down production so manufacturers’ priority is usually to minimize erroneous detections with acceptable detection rate. The detection rate can be improved, at the cost of the erroneous detection rate, during the configuration of the method or by replacing the Neural Classifier for a filter based on width/length.

The processing of the images has been carried out in such a way that it meets the real-time requirements of the current industries. This ensures that neither the acquisition nor the processing slows down the production, acquiring and processing 1 m of rail per second.

These results have been compared with two surface inspection systems bettering their performance for both defect detection rate and erroneous detections rate. Specifically, the results show and improvement on the defect detection rate of 26% and 17% respectively while the erroneous detections rate was reduced in 21 and 2 erroneous detections per product respectively. The reduction of the erroneous detection rate leads to less time wasted by the quality inspectors checking the detections while the improvement on the defect detection rate leads to less cost losses for the manufacturer.

A rail pattern with manually produced defects has been used as validation. This is a common validation procedure that can be performed each time the manufacturer adjusts the system in a systematic way. The results of this validation meet the ones obtained using the set of experimental rails proving that the method is efficient and fulfills the requirements of the current industry.

## Figures and Tables

**Table 1:** Step description and configuration.

Step	Description	Configuration
Median Filter	Section 2.3.1	Section 3.2
Segmentation	Section 2.3.2	Section 3.3
Bayesian Classifier	Section 2.3.3	Section 3.4
Neural Classifier	Section 2.3.4	Section 3.5

**Table 2:** Result comparison in the knowledge base.

Metric	Proposed Method	System A	System B
Recall	0.63	0.37	0.46
FP per Rail	6	27	8

**Table 3:** Defects in the rail pattern.

Number	Distance from Start	Depth	Width	Length	Detected before NN	Detected after NN
1	1324 mm	0.7 mm	0.5 mm	30 mm	X	-
2	1324 mm	0.8 mm	0.5 mm	30 mm	X	-
3	1530 mm	0.8 mm	0.75 mm	20 mm	X	X
4	1530 mm	0.9 mm	0.5 mm	20 mm	X	X
5	1824 mm	0.5 mm	0.5 mm	30 mm	X	X
6	1824 mm	0.5 mm	0.5 mm	30 mm	X	X
7	1928 mm	0.7 mm	0.5 mm	16 mm	X	X
8	1928 mm	0.5 mm	0.5 mm	20 mm	X	-
9	2500 mm	0.7 mm	0.5 mm	8 mm	X	X
10	2800 mm	0.8 mm	0.25 mm	16 mm	-	-
11	3000 mm	0.4 mm	0.75 mm	18 mm	X	X
